# Awareness of somatisation disorder among Swedish physicians at emergency departments: a cross-sectional survey

**DOI:** 10.1186/s12888-024-05652-z

**Published:** 2024-03-21

**Authors:** Carina Iloson, Marcus Praetorius Björk, Anna Möller, Karin Sundfeldt, Susanne Bernhardsson

**Affiliations:** 1 Department of Obstetrics and Gynecology, Institute of Clinical Science, Sahlgrenska Academy, University of Gothenburg, Gothenburg, Sweden; 2 Region Västra Götaland Competence Centre on Intimate Partner Violence, Gothenburg, Sweden; 3 Masthugget Gynaecology and Obstetrics Clinic, Region Västra Götaland, Gothenburg, Sweden; 4 Research, Education, Development and Innovation, Region Västra Götaland, Primary Health Care, Gothenburg, Sweden; 5 General Practice/Family medicine, School of Public Health and Community Medicine, Institute of Medicine, The Sahlgrenska Academy, University of Gothenburg, Gothenburg, Sweden; 6 Department of Clinical Science and Education, Karolinska Institutet, Stockholm, Sweden; 7 Department of Obstetrics and Gynecology, Stockholm South Hospital, Stockholm, Sweden; 8 Region Västra Götaland, Department of Gynecology, Sahlgrenska University Hospital, Gothenburg, Sweden; 9 Department of Health and Rehabilitation, Institute of Neuroscience and Physiology, Unit of Physiotherapy, Sahlgrenska Academy, University of Gothenburg, Gothenburg, Sweden

**Keywords:** Somatisation, Awareness, Physicians, Emergency

## Abstract

**Background:**

Somatisation is a highly prevalent psychiatric syndrome in both women and men, in which psychological distress is manifested in physical symptoms without a medical explanation. Many patients with somatisation disorder are high healthcare utilisers, particularly at emergency departments. Unnecessary investigations and diagnostic operations occur frequently, which cause both patient suffering and a significant burden on the healthcare system. Emergency department physicians’ awareness of somatisation and its manifestations has not previously been studied. This study aimed to investigate awareness about somatisation disorder among physicians working at emergency departments in western Sweden, and to explore differences between gender, specialty, and work experience.

**Methods:**

A web-based, cross-sectional survey consisting of six dichotomous questions about somatisation disorder was conducted, in December 2021 – January 2022, among licensed physicians of various specialties working at emergency departments in western Sweden. Descriptive analyses and comparative analyses were performed to investigate differences between gender, type of specialty, and years of practice. Data were analysed using chi^2^ tests and Fisher’s exact test.

**Results:**

Of the 526 eligible physicians who received the survey, 241 responded; response rate 45.8%. The majority of the respondents (56.4%) were women, and most (35.3%) were specialised in obstetrics/gynaecology. Average years of work experience was 11.1 (SD 8.7) years. Although 71% of respondents were aware of the diagnosis, only 7% knew the diagnostic criteria and only 6% had ever diagnosed a patient with somatisation disorder. Female physicians were more aware of underlying factors than their male colleagues (55.7% vs. 38.2%; *p* = .010). Type of specialty or years of practice did not affect awareness.

**Conclusions:**

Awareness of somatisation disorder is low among physicians working at emergency departments in western Sweden. The findings suggest a need to increase awareness and knowledge and provide training in diagnosing the condition, to ensure correct decisions and optimal patient management. Clinical guidelines need to be developed to support diagnosis, investigation, and treatment, in Sweden as well as internationally.

## Introduction

Somatisation is a psychiatric disorder in which a patient experiences and expresses psychological anxiety, presenting as physical (somatic) symptoms [1]. There are several terms with essentially the same meaning as somatisation: somatisation disorder, somatisation syndrome, somatoform disorder, conversion disorder/syndrome, and medically unexplained symptoms. In this study, we were interested in the diagnosis somatisation disorder. We therefore use the ICD-10-SE term “Somatisation disorder” [2], unless we refer to an article where another defined term is used. When the term is not defined in the studies, the general term “somatisation” is used.

Prevalence of somatoform disorders, including somatisation disorder and other medically unexplained somatic symptoms, ranges from 10 to 24% in young and middle-aged populations [3, 4]. Gender distribution of somatisation is scarcely reported but was in two studies found to be fairly equal in Qatar [3], and equal or slightly higher among men in Denmark [3, 4].

Many patients with somatisation symptoms are high utilisers of health care [5]. They seek more specialist care than primary care and have more emergency visits than non-somatising patients [6]. Patients undergo excessive investigations and repeated diagnostic surgeries without any medical explanation of the symptoms being identified [7]. This dilemma has been described as both a diagnostic and a healthcare cost problem internationally [8–10].

Patients experience the symptoms as real, and the condition is not similar to simulation. As these individuals seek emergency care, physicians at the emergency department have a unique opportunity to identify this condition early. A correct diagnosis may have a positive effect on the patient’s prognosis and healthcare costs.

Adult somatisation has been linked to both childhood and adulthood trauma, including sexual trauma, in both men and women [11]. Sexual abuse has been associated with patients having multiple somatic diagnoses [12]. In women, sexual trauma has been shown to affect somatisation more than nonsexual trauma [13]. A recent review has shown a link between sexual abuse and somatic symptoms [14]. Screening for underlying factors could facilitate identification of somatisation disorder as a possible diagnosis for these patients. Psychiatry professionals are well skilled in identifying and treating somatisation while medical doctors generally are less aware of somatisation disorder and, therefore, rarely diagnose the condition [15]. The Swedish healthcare system, like the ones in many other countries in Europe, is generally structured in such a way that somatic and psychiatric care are separated. Medical investigations are carried out either with a somatic or a psychiatric focus, but rarely both at the same time. This means that the psychiatric diagnosis of somatisation disorder often is handled by non-psychiatric physicians who generally do not have the in-depth knowledge about this diagnosis. In a survey among psychiatric and non-psychiatric physicians in the United States and the United Kingdom, some physicians reported that patients with a somatoform disorder were relatively rare in their practices (0–2%), while others estimated a high prevalence (> 20%) [16]. One third of the physicians stated that diagnostic guidelines were unclear. No studies have been identified on awareness among physicians who meet these patients in the emergency departments.

The primary aim of this study was to investigate awareness of somatisation disorder among physicians working in emergency departments in western Sweden. A secondary aim was to explore differences between gender, specialty, and work experience in physicians’ awareness about somatisation disorder. We did not expect any gender differences as education and practice are the same for men and women in Sweden. On the other hand, we hypothesised that experience provides higher awareness.

## Methods

### Study design and setting

A web-based cross-sectional survey was conducted among physicians working at emergency departments in five hospitals across western Sweden from December 2021 to January 2022. One was a large university hospital in a larger city (population 604,000) while the other four were medium sized hospitals in smaller cities (populations 58,000 to 115,000) [17].

### Participants

Eligible participants were licensed physicians of various specialties who had served as on-call physicians in emergency departments during the past 12 months. In Sweden, physicians of different specialties, such as internist, surgeon, orthopaedist, obstetrician/gynaecologist and emergency physicians all perform on-call services at emergency departments at regular intervals.

### Procedure

A brief web-based questionnaire, consisting of six dichotomous questions about somatisation disorder, was constructed. In addition to these variables, the following three demographic variables were constructed: gender, years of work experience, and type of specialty. An initial eligibility question asked whether the physician had served in the emergency department during the past 12 months, and only those who answered “yes” could continue to complete the survey. The questionnaire was constructed for this survey, with a strong emphasis on being brief and easy to respond to. Items were generated and refined within the research group. Content validity of the questionnaire was addressed by examining whether it was sufficiently comprehensive to cover the most important aspects of somatisation and ensuring that it contained no irrelevant items.

A draft of the questionnaire was pilot tested in a sample of 47 emergency physicians in another region of Sweden. For this pilot test, questions were added in which the respondents could share their views on whether and how the questions were difficult to understand, and whether they had any other comments on the questionnaire. Completion times of the survey were logged and examined. The pilot testers’ comments were reviewed by the research group, who reached consensus concerning clarifications and slight rephrasing of some items. Results from the pilot testing also formed the basis for a power calculation. Based on this, a sample size estimate of 133 was expected to provide 80% power, effect size w = 0.2, and 95% confidence interval.

Permission to distribute the survey was requested from clinic managers at the five largest hospitals in Region Västra Götaland, and email addresses of all licensed physicians at their clinics were obtained. An email was sent to the physicians with information about the study and an invitation to participate in the survey, accessible via a link in the email. Participants responded online, and the survey software **esMaker** (Entergate, 2018, Halmstad, Sweden) logged the responses and added them to a result database. Three reminder notices were sent via email at 1-week intervals.

All questionnaires were filled out anonymously and the answers could not be traced back to the respondents. A statement in the information email informed the respondents that participation was voluntary and that responding to the survey constituted their informed consent.

### Variables

#### Outcomes

Outcomes in the study were different aspects of awareness about somatisation disorder, measured through six different questions (Table 1). All six questions had yes or no as the only response options.

**Table 1:** Questions asked to physicians about *Somatisation disorder F45.0* (ICD-10-SE) at six emergency departments in western Sweden

Question*	Response
**Question for eligibility**: Have you worked as licenced physician at an emergency department during the last 12 months?	Yes/No
Are you aware of the diagnosis Somatisation disorder F45.0?	Yes/No
Do you know the diagnostic criteria for the diagnosis?	Yes/No
Have you ever registered the diagnosis?	Yes/No
Have you ever treated a patient that you knew had the diagnosis?	Yes/No
Have you ever treated a patient that you suspected had the diagnosis?	Yes/No
Do you know anything about underlying factors?	Yes/No
Number of years working as a licenced physician?	A number
Current specialty?	Five options
Gender?	Woman/Man/Other

*The questions have been translated from Swedish

#### Covariates

The three variables gender, years of work experience, and specialty were used as covariates in the analysis. The variable *Gender* included the response options man, woman, and other, but was in the analysis treated as a dichotomous nominal scale of men and women, because no respondent defined themselves as non-binary. The variable *years of experience* was in the survey designed as a continuous variable (to allow calculating mean and standard deviation) and was thereafter coded as an ordinal scale of 0–5 years, 6–10 years, 11–15 years, 16–20 years and over 20 years of experience. The variable *specialty* had five response options: internal medicine, surgical, orthopaedic, obstetrics/gynaecology or emergency. Due to too few participants in the emergency specialty (*n* = 14), internal medicine and emergency were coded into a merged specialty labelled “medicine”. This resulted in a nominal scale of a total of four specialties: medicine, surgery, orthopaedics, and gynaecology.

### Statistical analyses

Differences in awareness about somatisation disorders were reported using frequencies and distributions and analysed using the Chi-square test of proportions and Fisher’s exact test. There were six questions measuring awareness about somatisation disorders. The overall proportion of *Yes* versus *No* answers were analysed using the Chi-Square Goodness of Fit Test. Covariates, including gender, work experience, and specialties were analysed using the two-way Chi-square test. Sub-group analyses of potential associations to specialty, gender, and years of work experience were performed using Fisher’s exact test. A *p*-value of ≤ 0.05 was considered statistically significant. All analyses were performed using IBM SPSS Statistics 28.0.

## Results

Of the 632 physicians invited to participate, 347 physicians (54.9%) answered the eligibility question in the questionnaire. Of those, 106 physicians (30.5%) did not meet the inclusion criteria, due to not having served in the emergency department during the past 12 months. In total, 241 of the eligible 526 physicians responded to the survey, yielding a response rate of 45.8% (Table 2). Internal missing values ranged from 0 to 2. In the event of internal missing values in specific questions, the participant was excluded from the analysis of that question. The majority of the respondents were women (56.4%). The average participant had 11.1 years of work experience (Sd 8.7; Md 8). Most respondents (35.3%) were from the obstetrics/gynaecology specialty.

**Table 2:** Characteristics of survey respondents (*n* = 241)

Variable	n	%
**Gender**		
Women	136	56.4
Men	105	43.6
**Work experience** (yrs)		
0–5	67	27.8
6–10	82	34.1
11–15	35	14.5
16–20	23	9.5
> 20	34	14.1
**Specialty**		
Internal medicine	26	10.8
Surgical	52	21.6
Orthopaedic	64	26.6
Obstetrics/Gynaecology	85	35.3
Emergency	14	14.1

### Awareness of somatisation disorder

A majority of the respondents (71.4%) reported being aware of the diagnosis somatisation disorder, F45.0 (ICD-10-SE), see Table 3. A large majority of respondents (92.9%) reported not knowing which diagnostic criteria are included in the diagnosis and claimed to have never diagnosed patients in practice (94.1%). Most respondents had never treated patients they knew had the diagnosis (65.7%), but most (68.0%) claimed they had treated patients they suspected had the diagnosis.

**Table 3:** Overall responses by emergency department physicians to six questions regarding the diagnosis *Somatisation disorder* F45.0 (ICD-10-SE)

Item and responses	n (%)*	Total
Awareness of the diagnosis	172 (71.4)	241
Knowledge about diagnosis criteria	17 (7.1)	241
Ever diagnosed in practice	14 (5.9)	239
Ever treated patient that you know had the diagnosis	82 (34.3)	239
Ever treated patient that you suspected had the diagnosis	166 (68.0)	240
Awareness about underlying factors of the diagnosis	115 (47.9)	240

^*^Corresponds to answering yes on the six questions regarding somatisation disorder F45.0 (ICD-10-SE)

### Differences between gender, specialties, and years of work experience

Although there were few gender differences among the physicians regarding awareness about somatisation disorders, a higher proportion of female physicians (55.1%) reported knowing about the underlying factors in somatisation than their male colleagues (38.5%; *p* = .010 Table 4). There were no significant differences between specialties nor years of work experience, with respect to awareness or use of the somatisation disorder diagnosis.

**Table 4:** Distribution of responses by gender, work experience and specialisation to six questions regarding the diagnosis *Somatisation disorder* (F45.0)

	Awareness of the diagnosis (N = 241)	Knowledge about the diagnostic criteria (N = 241)	Ever diagnosed in practice (N = 239)	Ever treated patient that you know had the diagnosis (N = 239)	Ever treated patient that you suspected had the diagnosis (N = 240)	Knowledge about underlying factors of the diagnosis (N = 240)
	n (%)^a^	n (%)^a^	n (%)^a^	n (%)^a^	n (%)^a^	n (%)^a^
Gender						
Women	101 (74.3)	11 (8.1)	7 (5.2)	48 (35.6)	98 (72.1)	75 (55.1)*
Men	71 (67.6)	6 (5.7)	7 (6.7)	34 (32.7)	68 (65.4)	**40 (38.5)**
Work experience (yrs)						
0–5	47 (70.1)	5 (7.5)	2 (3.0)	23 (34.3)	44 (65.7)	30 (44.8)
6–10	56 (68.3)	2 (2.4)	2 (2.5)	32 (39.5)	57 (70.4)	35 (42.7)
11–15	24 (68.6)	3 (8.6)	1 (2.9)	8 (22.9)	25 (71.4)	17 (48.6)
16–20	19 (82.6)	3 (13.0)	5 (21.7)	8 (34.8)	16 (73.9)	12 (52.2)
>20	26 (76.5)	4 (11.8)	4 (11.8)	11 (33.3)	23 (67.6)	21 (63.6)
Specialty						
Medicine	32 (80.0)	3 (7.5)	1 (2.6)	20 (50.0)	29 (74.4)	19 (47.5)
Surgical	38 (73.1)	6 (11.5)	4 (7.7)	20 (39.2)	36 (69.2)	24 (47.1)
Orthopaedics	48 (75.0)	2 (3.1)	3 (4.8)	18 (28.6)	45 (70.3)	31 (48.4)
Obstetrics/Gynaecology	54 (63.5)	6 (7.1)	6 (7.1)	24 (28.2)	56 (65.9)	41 (48.2)

^a^Corresponds to answering “yes” to the six questions regarding somatisation disorder F45.0 (ICD-10-SE). *Note*: Chi-square test: significant results are marked with * (*p* = .010). In cells with less than 5 numbers, analysis was not conducted

### Gender differences within specialties regarding awareness of somatisation disorder

**Fig. 1:**
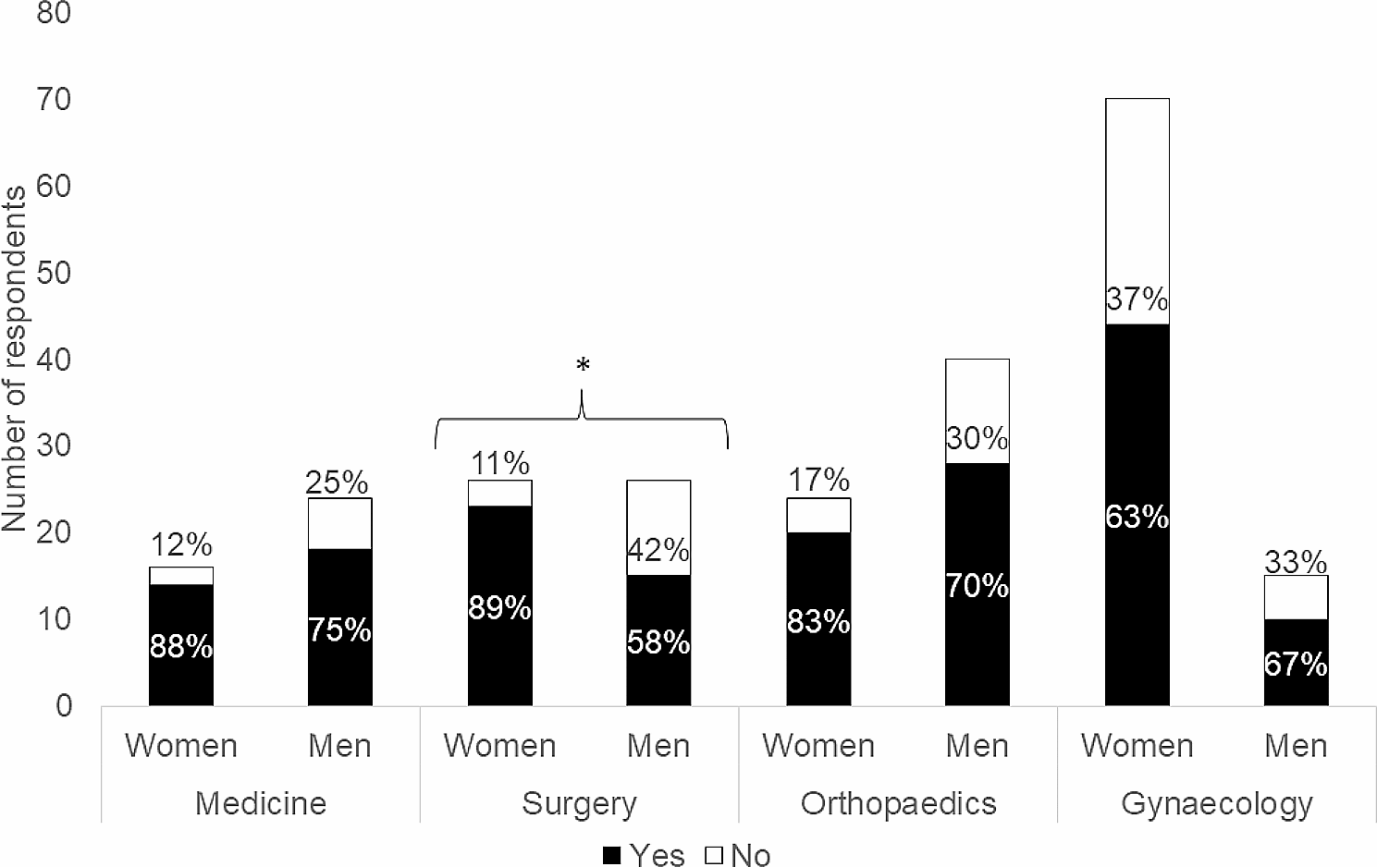
Subgroup analyses of gender and specialisation regarding awareness of the diagnosis Somatisation disorder F45.0 (ICD-10-SE). *Note*: Chi-square test: significant results are marked with * (*p* = .027)

Of respondents specialising in surgery, more women (88.5%) reported being aware of the diagnosis, compared with men with the same specialty (57.7%; *p* = .027, Fig. 1).

**Fig. 2:**
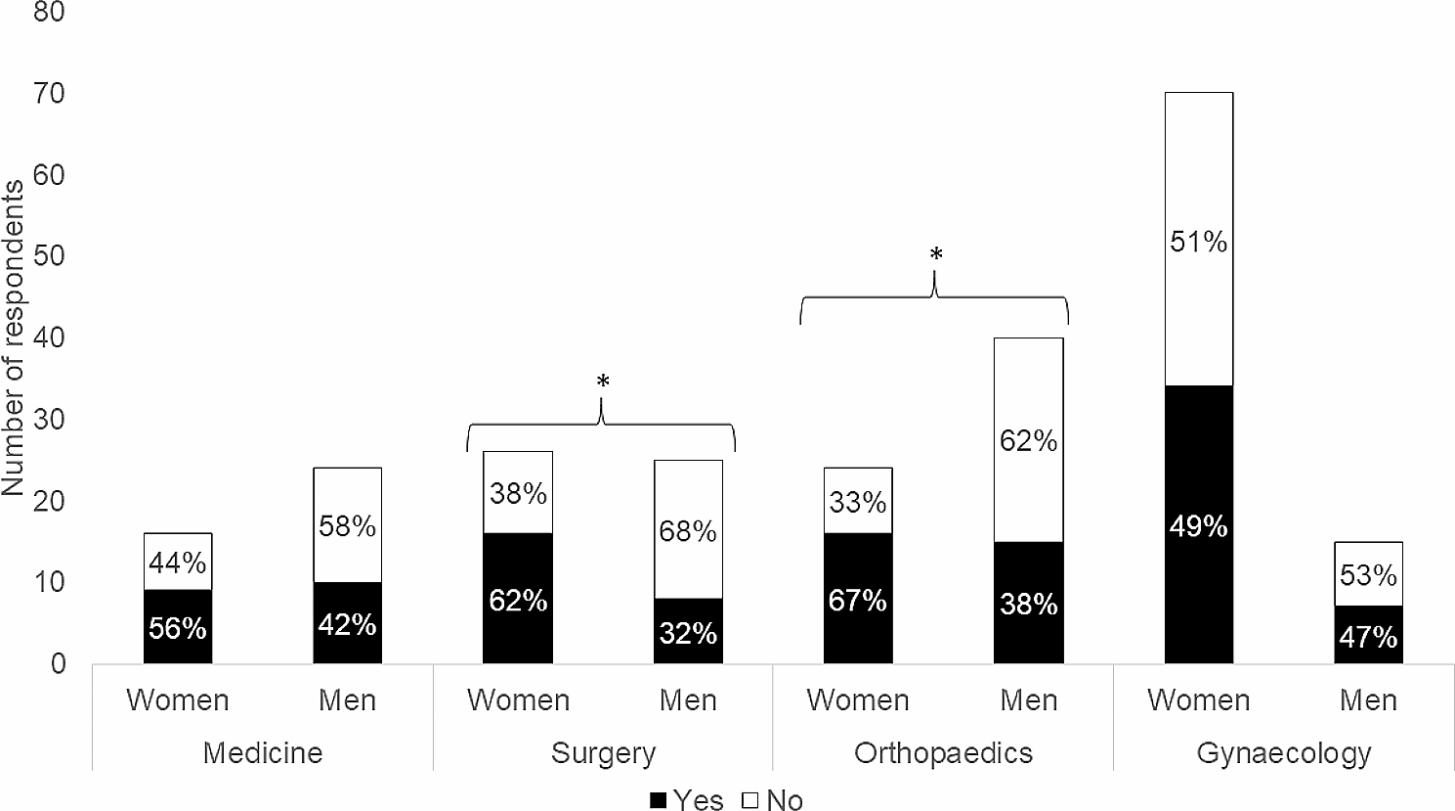
Subgroup analyses of gender and specialisation regarding awareness of the underlying factors of the diagnosis Somatisation disorder F45.0 (ICD-10-SE). *Note*: Chi-square test: significant results are marked with * (*p* = .050; *p* = .038)

More women specialised in surgery (61.5%) also stated that they knew about the underlying factors of somatisation compared with men (32.0%) of the same specialty (*p* = .050, Fig. 2). Women specialised in orthopaedics (66.7%) stated that they knew more about the underlying factors of somatisation compared with men (37.5%) of the same specialty (*p* = .038, Fig. 2).

## Discussion

The study aimed to investigate awareness of somatisation disorder among physicians working in emergency departments in western Sweden. The study showed that the level of knowledge regarding somatisation disorder is low, across disciplines. There were generally few differences between the genders, but female physicians were more aware of underlying factors to somatisation disorder than their male colleagues, while type of specialty or years of practice did not affect awareness.

As many as one fifth of the population suffers from some type of somatoform disorder [3] and the probability of meeting this patient group as a physician at an emergency department is high. The low level of awareness about somatisation disorder, its diagnostic criteria, and underlying factors shown in our survey, entail a risk for failure to identify this condition. Such a failure may cause the unwary physician to initiate investigations or diagnostic procedures that may result in iatrogenic complications and considerable financial burden to the society.

A single underlying explanation for somatisation can be difficult to find. The suggested link between violence, especially sexual violence, and somatisation [13, 14] underscores the importance of being aware of the condition so that it can be identified among the women who seek emergency care for symptoms that could be related to somatisation. Several studies have reported associations between somatic symptoms and sexual abuse [18–20]. Emergency departments provide a unique opportunity for healthcare professionals to screen patients for intimate partner violence [21], which could increase the possibility of earlier identification and diagnosing of somatisation disorder.

Physicians seem to find somatoform disorders extremely challenging to describe in both clarity and utility [16]. In our study, three of ten responding physicians stated that they had no awareness of somatisation disorder. Nine of ten respondents reported not knowing the diagnostic criteria for the diagnosis. This low level of awareness seemed to be similar across disciplines, suggesting that none of the involved specialties that work in emergency departments are better equipped than others to identify and manage patients that potentially may suffer from somatisation disorder. Despite this, nearly seven of ten physicians claimed they had treated patients they suspected had the diagnosis. As physicians’ awareness and knowledge about somatisation disorder is poorly researched, comparing our findings to other studies was challenging. We found a few studies on physicians’ knowledge about other difficult to diagnose conditions. Our findings are in line with a Canadian survey of physicians’ knowledge of fibromyalgia, in which physician knowledge of fibromyalgia diagnostic criteria was poor and linked to specialist training [22]. Similarly, an Indian study described how family and primary physicians had trouble separating anxiety, depression and somatic presentations amongst their patients [23], pointing to the difficulty in establishing a correct diagnosis and proceed with optimal patient management. In contrast, a Dutch study on family physicians’ recognition of medically unexplained physical symptoms, a condition close to somatisation, showed that the participants believed they could properly identify the condition in their patients [24].

Our expectation was that increased experience would lead to increased awareness, as shown in a study from Saudi Arabia that assessed paediatric physicians’ knowledge of febrile seizures [25]. That study showed that the consultants, with more years of practice, had better knowledge about febrile seizures in comparison to other groups of physicians. However, our study showed no significant differences in awareness related to years of work experience. The previously mentioned Canadian study, which investigated knowledge about fibromyalgia, also found that knowledge was independent of clinical experience [22]. It is difficult to explain why years of experience with these diagnoses do not increase the level of knowledge, which one could expect. Experience in terms of years of practice and its association to knowledge and performance seems rather complex. Although clinical experience can lead to increased clinical knowledge [25], a systematic review from 2005 showed that clinical experience was negatively related to physicians’ quality of care [26]. A possible explanation for this could be that medical advances occur frequently, entailing a risk that the knowledge that physicians possess may become outdated. Therefore, it is possible that greater experience does not lead to increased knowledge in the context of somatisation, which has a history of a lack of diagnostic criteria and an unclear definition of the disorder. However, both the 2013 revised DSM 5 and the new ICD-11 from 2019 have simplified the diagnosis and may possibly improve these weaknesses in the future. In this study, however, we used ICD-10-SE, as ICD-11 has not yet been translated into Swedish.

A previous study has proposed to generally increase physicians’ competence [27]. In addition, we suggest engagement in deliberate practice, with training focused on improving specific tasks, in this case, related to somatisation disorder. This could lead to better knowledge and performance in this field. It is well known that knowledge is essential to make better decisions and judgments [28]. Furthermore, the working environment in emergency departments, with quick decisions and seriously ill patients together with a complex condition such as somatisation, is challenging and may also explain the lack of effect of experience. Under these circumstances, focusing on individual symptoms may be a pragmatic solution and guide the physicians’ investigation, which may be another explanation for not developing increased knowledge about somatisation despite increased years of practice.

In our study, women reported knowing more about the underlying factors of the somatisation disorder than their male colleagues. Also, there were gender differences in awareness of somatisation disorder within specific specialties, consistently in favour of the female physicians. In contrast, a study from Wisconsin, USA, reported that female physicians consistently assessed their ability to perform or apply knowledge and skills related to clinical research lower than how men ranked themselves [29]. It is, however, suggested that gender differences in self-perception of abilities and competence are related to gender-specific tasks. In a study by Beyer and Bowden [30], participants were asked to rate specific tasks as either “feminine” or “masculine”, and thereafter perform the tasks and finally rate their confidence of their own performance. In masculine specific tasks, women self-evaluations of their own performance tended to be inaccurately low in relation to their actual performance. This was not seen in feminine specific tasks. It is possible that knowledge about somatisation disorder is more of a “feminine task”. A systematic review, conducted in 2013 [31], reported greater patient engagement by female doctors and some evidence to suggest that female physicians adopt a more partnership-building style and spend an average of 2.2 min longer with patients per consultation than their male colleagues. This communication style may enable disclosure of underlying factors such as intimate partner violence and increase knowledge among female physicians about somatisation in general.

An observational cohort study from Canada [32], identified a similar communication style, patient-centred practice, to increase the efficiency of care by reducing diagnostic tests and referrals. Unnecessary investigations and diagnostic operations that are common in somatisation patients could hence be reduced. Despite the gender differences described above, it can be noted that for most questions, men and women in our study answered relatively similarly regarding awareness and use of the somatisation disorder diagnosis.

This study has several limitations. The questionnaire was developed specifically for the study and was only preliminary validated. However, the pilot test, conducted in a similar cohort of physicians in a different part of Sweden, indicated both content and face validity of the questionnaire. Furthermore, it was not constructed as a psychometric tool, but rather as an indicator of awareness. The responses are self-reported, increasing the level of uncertainty and may also, as all self-report data, reflect a social desirability bias. The results of the self-administered web-based survey are subject to non-response bias, which may result in overestimation of awareness. The inclusion criteria of having served in an emergency department during the past 12 months may entail some variability in the extent of the respondents’ experience. Drawn from only a handful of hospitals in Sweden, caution must be used in generalising the findings to other countries. However, the problem of suboptimal clinical management of patients with somatisation has been identified in other countries [8–10], and we believe the knowledge gap we identified in this sample of Swedish physicians is relevant for other countries, especially those with similar healthcare systems.

The main strength of this survey is that it consists of six dichotomous questions, making it easy and rapid to complete, even for very busy physicians. Dichotomous response options force the respondent to choose an alternative – but may also not reveal the nuances that more response options potentially could have provided. The questionnaire reached a large study population with almost 50% response rate. We believe this survey of physicians’ awareness can provide a basis for future research and that it also can be useful to inform a future design of an intervention to increase physicians’ knowledge in this important field. Further research to replicate our findings in other countries is warranted. Future studies are also proposed to take a closer look at how physicians are trained in diagnosing somatisation disorder, and perhaps new clinical guidelines, standards, CME points, etc. are warranted to support diagnosis, investigation, and treatment, both in Sweden and internationally. In addition, the patient’s perspective needs to be highlighted, such as in a study of patients’ experience of consultations with physicians at the emergency departments before being diagnosed with somatisation disorder.

In conclusion, the level of awareness about somatisation disorder is low among physicians working at emergency departments in western Sweden. Three of ten emergency doctors stated that they had no awareness of somatisation disorder. Patients who meet doctors who lack awareness of the disorder are at risk of unnecessary investigations and treatments. The findings suggest a need to increase awareness and knowledge amongst physicians and provide training in diagnosing the condition, to ensure correct decisions and optimal patient management. Correct diagnosis entails substantial benefits in terms of more adequate treatment for the patient and more efficient use of resources in health care and society.

## Data Availability

The datasets analysed during the current study are available from the corresponding author on reasonable request.
